# Effect of vaccination on the use of antimicrobial agents: a systematic literature review

**DOI:** 10.1080/07853890.2020.1782460

**Published:** 2020-06-29

**Authors:** T. Mark Doherty, William P. Hausdorff, Karl G. Kristinsson

**Affiliations:** a Global Medical Affairs, GSK, Wavre, Belgium; b PATH, Washington, DC, USA; c Faculty of Medicine, Université Libre de Bruxelles, Brussels, Belgium; d Department of Clinical Microbiology, Landspitali University Hospital, Reykjavík, Iceland; e Faculty of Medicine, University of Iceland, Reykjavík, Iceland

**Keywords:** Vaccination, antimicrobial resistance, antimicrobial use, meta-analysis

## Abstract

**Background:**

Antimicrobial resistance is a growing global health threat. To preserve the effectiveness of antimicrobials, it is important to reduce demand for antimicrobials.

**Objectives:**

The objective of the study was to screen the existing peer-reviewed literature to identify articles that addressed the potential impact of influenza or *Pneumococcus* vaccination on antibiotic usage.

**Data sources:** PubMed, Embase

**Study eligibility criteria:** Clinical studies where antimicrobial prescribing was assessed in both vaccinated and unvaccinated populations.

**Participants and interventions:** All patient populations were included (infants, children, adults and elderly), where the effects of the intervention (vaccination) was assessed

**Results:**

We identified unique 3638 publications, of which 26 were judged to be of sufficiently high quality to allow the calculation of the potential impact of vaccination. Of these studies 23/26 found a significant reduction in antibiotic use by at least one of the parameters assessed.

**Limitations:**

Different measures used to define anti-microbial use, studies typically focus on specific risk groups and most studies are from high-income countries.

**Conclusions and implications of key findings:** Despite the limitations of the review, the evidence indicates that improved coverage with existing vaccines may significantly reduce antimicrobial demand. This suggests it may be a valuable tool for antimicrobial stewardship.

**Key messages**
- While vaccines against a number of pathogens have been studied for their ability to reduce antimicrobial use, currently only vaccination against influenza or pneumococcus has generated sufficient data for analysis
- Vaccination against either influenza or pneumococcus significantly reduced overall antimicrobial prescribing rates, both in vaccinated individuals and at a population level
- Maintaining and expanding vaccination coverage thus appears to be a key tool for antimicrobial stewardship

## 1. Introduction

Alongside the provision of clean drinking water and vaccination, antimicrobials stand as one of the most important health interventions of the last two centuries and have helped dramatically reduce both morbidity and mortality from infectious disease [1]. The prospect of reduced effectiveness of antimicrobial treatment due to the spread of pathogens resistant to most antimicrobial agents (AMR) is therefore of great concern [2]. This is not a new problem: the problem of evolving AMR was discussed in detail by Falkow over 40 years ago [3]. However, an increasing number of reports bears witness to a steep increase in the prevalence of AMR, both in terms of the number of different pathogens affected, in the proportion of AMR isolates and the emergence of strains resistant to treatment with almost all existing antimicrobials [4]. There are already reports of strains which are essentially untreatable by existing programmes or nearly incurable with any antimicrobial, moving this issue from academic debate to burgeoning medical emergency [5,6]. Multiple reviews from groups as diverse as the World Health organization (WHO), the World Bank, the UK government, the European Commission and the European Centre for Disease Prevention and Control (ECDC), have produced a consensus that AMR provides a real and growing threat to human health. There is also agreement that improved antimicrobial stewardship (the preservation of the clinical utility, as far as is possible, of existing antimicrobials), as well as development of new classes of antimicrobials, is urgently needed [2,4].

Multiple antimicrobial stewardship activities are already underway, but these have primarily focussed on what could be described as curbing inappropriate exposure to antimicrobials – just a few examples include reducing or eliminating antimicrobials in wastewater, reducing use in agriculture, better prescribing guidelines for clinicians and more rapid access to diagnostic tools to guide prescribing [2]. It is also understood that use of new antimicrobials should be restricted, where possible, to prolong their useful clinical life [2]. There is, however one key concept – discussed by Falkow and other researchers [1,3], but still often underappreciated by policy-makers and the public at large – which is that the development of AMR is a predictable and essentially inevitable outcome of the use of antimicrobials, even when they are used responsibly. In many cases, physicians are forced to initiate treatment with antimicrobials empirically – i.e. on the basis of symptoms, without diagnostic confirmation of the identity or antimicrobial susceptibility of the presumed pathogen – simply because the consequences of delaying treatment can be severe [2]. Thus, even in well-resourced countries such as the USA, where diagnostic tools are readily available, it has been estimated that as many as 67% of all antimicrobial prescriptions for respiratory infections are inappropriate [7] while a UK analysis suggested that over a third of all antibiotic prescriptions had no clinical justification [8]. The same is true for other infections as well, e.g. for treating diarrheal diseases despite evidence that frequently it may not be clinically appropriate [2]. This overuse of antimicrobials for humans and animals has serious consequences as some important pathogens are becoming resistant to the majority of commonly used antimicrobials (extensively drug-resistant) or even all antimicrobials (pan drug-resistant) [9,10].

While better rapid diagnostics may be able to reduce inappropriate antimicrobial prescribing in clinical settings, the risk of resistance developing remains, even where the use of antimicrobials is clinically justified. As a simple example, multiple countries have reported an increasing proportion of AMR isolates from patients diagnosed with acute otitis media (AOM) [11]. This is particularly problematic, since antimicrobials are the primary tool for treatment of AOM, and widespread, albeit appropriate, use of β-lactam antibiotics for AOM is thought to have contributed to the development of AMR [11]. It is therefore a reasonable simplification, to say that *any* use of antimicrobials may potentially contribute to the emergence of AMR. Reducing the incidence of symptomatic disease requiring antimicrobial treatment – for example, by vaccination – can play a role in reducing antimicrobial use and thus, by implication, retard the development of AMR. However, for authorities to integrate vaccination into their plans to reduce the spread of AMR pathogens, a better oversight of the costs and benefits is needed which may be hindered by the current lack of reliable data on which to base concrete policy recommendations [2]. This systematic literature review therefore attempts to assess current data on the impact of vaccination on antimicrobial usage and provide an estimate of what could be achieved by improving vaccine coverage.

### 1.1. Objectives

The objective of this study was to screen the existing literature to assess the effect of vaccination on the use of antimicrobial agents. While a broader screening strategy was initially trialled, the current analysis focuses on vaccination against influenza and *pneumococcus*, the only two vaccines for which a significant body of data is currently available.

**Figure 1.**
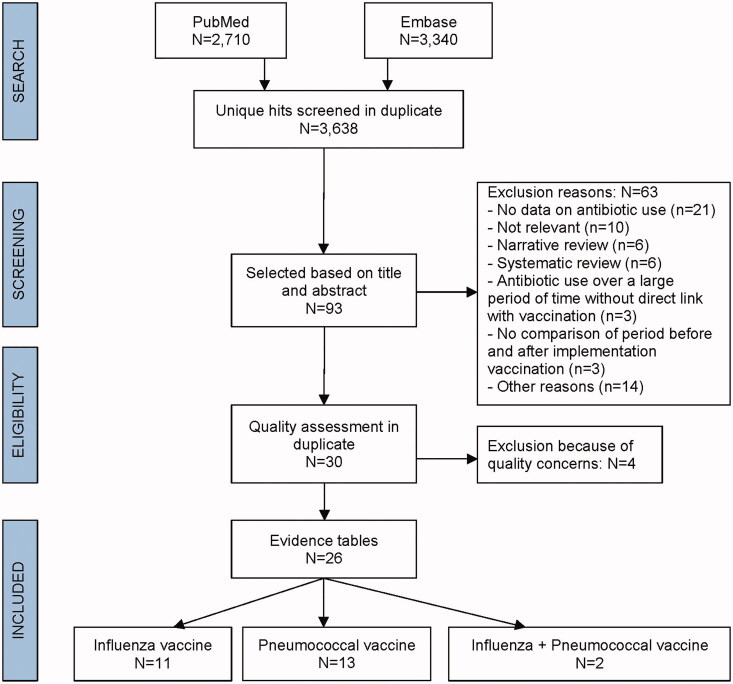
CONSORT diagram.

## 2. Materials and methods

### 2.1. Selection criteria

Since no listings of prior systematic reviews on this topic could be identified using Cochrane’s PROSPERO tool when the review was initiated, no existing review protocol number is reported here. The authors therefore developed the protocol with the collaboration of Pallas health research and consultancy (www.pallashrc.com) before conducting the literature search. Before completing the full analysis presented in this review, initial search strategies were tested on PubMed, and these did not include a restriction on pathogens (PICO Intervention parameter, Search string #2, shown below). This initial screening indicated a number of vaccine-preventable infections (for example, *Haemophilus influenzae*, measles and rotavirus) for which potentially relevant studies have been published. However, with the exception of influenza and pneumococcal vaccination, only 1 or 2 studies were identified, rendering a systematic analysis of those vaccines essentially meaningless. Therefore, the analysis was restricted to influenza and pneumococcal vaccination, for which a substantial number of studies have been published. In addition, it was noted in the initial screening searches restricted to broad antimicrobial terms such “antibiotic” did not always identify publications where specific antimicrobials were studied. Therefore, specific terms for antimicrobials prescribed for infections associated with influenza or pneumococcal were included in the search strategy. Finally, since the objective was to analyse clinical data with the goal of supporting healthcare-related decision-making, animal studies were excluded. The analysis covers publications in the period 01 January 2000 to 01 October 2018, and was restricted to publications in English, French, Dutch, Danish and Icelandic, so that original content could be directly assessed by the authors. The search covered both Medline (accessed *via* PubMed) and Embase, returning 2710 hits and 3340 hits respectively.

### 2.2. Search strategy

Search categories and PICO headings

- **P**opulation (Human, all, dates, 01 January 2000 to 01 October 2018)
- **I**ntervention (Vaccination against pneumococcus and/or influenza)
- **C**omparison (Vaccinated/Unvaccinated study populations)
- **O**utcomes (Antimicrobial use, any methodology).

The following strings were used:

**#1 vaccination**

vacc*[tiab] OR immun*[tiab]

**#2 diseases**

pneumococc*[tiab] OR influenza*[tiab] OR flu[tiab]

**#3 antimicrobials**

antibiotic*[tiab] OR antimicrobial*[tiab] OR aminoglycoside[tiab] OR amoxicillin*[tiab] OR ampicillin*[tiab] OR azithromycin*[tiab] OR β-lactam*[tiab] OR carbapenem*[tiab] OR cefotaxime[tiab] OR ceftriaxone*[tiab] OR cephalosporin*[tiab] OR chloramphenicol*[tiab] OR ciprofloxacin*[tiab] OR clavulan*[tiab] OR clindamycin[tiab] OR cotrimoxazole[tiab] OR co-trimoxazole[tiab] OR erythromycin*[tiab] OR fluoroquinolone*[tiab] OR gentamicin*[tiab] OR gentamycin* [tiab] OR penicillin*[tiab] OR piperacillin*[tiab] OR quinolone*[tiab] OR streptomycin*[tiab] OR sulfamethoxazole[tiab] OR tetracyclin*[tiab] OR trimethoprim*[tiab]

**#4 animal studies**

animals[Mesh] NOT (humans[Mesh] AND animals[Mesh])

The search strategy for PubMed was (#1 AND #2 AND #3) NOT #4, while for Embase it was (#1 AND #2 AND #3)

### 2.3. Selection of articles

To reduce as much as possible any selection or publication bias, the articles to be reviewed were identified by a two-step selection protocol devised by an independent health research and consulting company, Pallas (www.pallashrc.com), commissioned specifically for the task by GSK (Figure 1). First, articles were retrieved from the PubMed and Embase databases. Then, after removal of duplicates and screening for relevance (see the PICO headings above), the retrieved articles were assessed using CONSORT guidelines to ensure all studies included were considered standards-compliant. The full list was screened based on title and abstract yielding potentially relevant articles to be assessed on the basis of their full text. The selection criteria were finalized prior to screening of articles. Articles were considered relevant if they discussed clinical studies where antimicrobial prescribing was assessed in both vaccinated and unvaccinated populations, either side by side or chronologically. All patient populations were included (infants, children, adults and elderly), and study designs included randomized controlled trials (RCTs), observational studies, and population-based database studies.

The exclusion criteria were that a study did not address the review objective, that it was not a primary epidemiologic or clinical study (case series, case reports, review articles and editorials were considered out of scope) or it represented a report in the so-called “Grey literature” such as meeting abstracts, letters, websites, etc. (Figure 1).

In the second selection step (also performed by Pallas), the full text articles selected in step 1 were assessed using two additional exclusion criteria. These were that the methods section did not provide sufficient details to fully assess the study population or methodology, or that no quantitative data could be retrieved from the article. As the goal was to assess the relationship between vaccination and antimicrobial use, six articles discussing antimicrobial use in a time period where vaccination was introduced, but which did not provide data on uptake of the vaccine or vaccination coverage data, were also excluded, since this rendered it impossible to quantitatively assess the impact of vaccination. While this (and the exclusion of the grey literature) raises the issue of incomplete retrieval of identified research, the inability of this material to provide quantitative insight into the question posed in the initial protocol means that they could not contribute to addressing this question, as published.

### 2.4. Risk of bias and quality of the study evidence

The analysis employed the methodology checklists from the Scottish Intercollegiate Guidelines Network (SIGN) [12]. The SIGN checklists include the criteria on publication quality and reference the PRISMA, STROBE and CONSORT guidelines. The PRISMA guideline is useful for critical appraisal of published systematic reviews, although it is a guideline for publication rather than a quality assessment instrument to gauge the quality of a systematic review and is not used for assessment of included studies. In a similar vein, the STROBE statement for observational studies provides a checklist for publication structures, including potential quality criteria, while the CONSORT statement includes a checklist of items that must be reported in papers describing RCTs in order to be considered compliant. The SIGN checklists (see Supplementary material 1) were used to identify publications with potential reporting or critical quality issues. For this, the predefined aspects of a study were qualitatively reviewed for each publication by checking compliance of the methodology against a checklist for each specific study design and assigning a response of “Yes”, “No”, “Cannot be determined” or “Not Applicable” to each question. At the end of this process, the evaluator rated the overall quality assessment of a study. There are four possible categories of study quality:

- High quality (++): When the majority of the criteria/questions in the SIGN checklist are met, the quality of the study is rated as high (++), which means that there is little or no risk of bias, and the results are unlikely to be changed by further research.
- Acceptable (+): Most criteria/questions in the SIGN checklist are met then the quality of the study is scored as acceptable (+), which means that some flaws in the study may be present with an associated risk of bias, and the study conclusions may change in the light of further studies.
- Low quality (−): Most criteria are not met, the study quality is rated as low (−) which means that there may be significant flaws to key aspects of the study design, and further research is very likely to change the study conclusions.
- Unacceptable – reject (0): The study quality is unacceptable and therefore the study was excluded.

The final decision whether the quality of a study was sufficient for inclusion was based on the expertise of the Pallas epidemiologists involved, based on the results of the SIGN checklist and the objectives of the review in mind. In case of doubt, studies were discussed with a second or third epidemiologist.

There is no SIGN checklist available for, or suitable for, scoring population-based database studies, so the ROBINS-I methodology was applied to these to these studies. The outcome in all cases was: “No information on which to base a judgement about risk of bias” based on the finding “There is no clear indication that the study is at serious or critical risk of bias and there is a lack of information in one or more key domains of bias (a judgement is required for this).” Therefore, it was decided to screen included studies for specific limitations, relevant for the purposes of this review, and include study details in the evidence tables (see Supplementary Materials 2). The following questions were asked:

- Does the study compare multiple years before and after introduction of the vaccine?
- Is there a link on individual level between vaccination data and antimicrobial use?
- Is the study large enough to have sufficient power?
- Is vaccination coverage data available for the population under investigation?

No studies were excluded based on this analysis.

### 2.5. Quality control

The following quality control measures were applied:

- All titles and abstracts were screened independently by two researchers from Pallas. The results were compared and discussed.
- All full text articles were critically appraised independently by two researchers from Pallas. The results were compared and discussed early in the process.
- Data extraction: the evidence tables were compiled by one senior researcher and checked by another senior researcher from Pallas. The selection was provided as a separate document, which was independently re-reviewed by all three authors
- Validation: all full-text articles were read independently by two of the authors (each paper was assigned randomly to two of the three authors of this manuscript), and compared against the compiled evidence tables provided by Pallas to ensure accuracy. A short summary was drawn up by each author in advance of a meeting where the articles and data tables were discussed in plenum. Any disagreement between two authors was adjudicated by the third.

## 3. Results

Of the 3638 potential articles identified after screening of the Medline and Embase databases and the elimination of duplicate entries, 93 were assessed as being of potential relevance based on title and abstract. The full text of these were therefore reviewed. Based on the procedures discussed in the methodology section, 30 articles were identified as potentially relevant for inclusion in this analysis. The RCTs and non-randomized studies were critically appraised using the SIGN checklists (Supplementary material 1). As there is no SIGN checklist for scoring population-based database studies, these were evaluated on study-specific limitations most relevant for the purposes of this literature review (as described in the methodology section). Four articles were excluded because of a low SIGN score and/or limited description of the study characteristics, which made quantitative assessment problematic [13–16]. The procedure and exclusion criteria are detailed in the flowchart in Figure 1 and the publications included for full analysis are presented in Table 1, broken down by study design and quality assessment. Study design, study population characteristics and the vaccines used in all studies included in this review are also detailed in Supplementary material 2. Extracted data were grouped by study type and quality and presented in the data tables, without further synthesis, meta-analysis or subgroup analysis. A simple narrative summary is presented in the Discussion.

**Table 1.** Studies included in the final analysis.

Study quality (SIGN)	Randomized controlled trials	Non-randomized studies	Population-based database studies
High quality (++)	[17–20]	[21,22]	
Acceptable quality (+)	[23–26]	[27–31]	
Low quality (−)		[32,33]	
Not possible to assess with SIGN (o)			[34–42]

SIGN: Scottish Intercollegiate Guidelines Network.

### 3.1. Influenza vaccination

Of the studies included, 11 presented data on influenza vaccination and antimicrobial use. The readout in the studies was varied, with antimicrobial use presented as number of antimicrobial prescriptions, courses or administration, number of days of oral antimicrobials, number of days of injected antimicrobials or whether patients had received antimicrobials. Four studies described antimicrobial use in children [17,23,27,34], one in mother-infant pairs and their household contacts [21], four in adults [28–30,32], one reported results from a population-level study comparing universal and targeted vaccination policies [31], and one in household contacts of vaccinated children [24].

The four studies examining antimicrobial use in vaccinated children (summarized in Table 2) varied significantly in quality, as assessed by SIGN guidelines. One RCT found a more substantial decrease (44%) in antimicrobial prescriptions among vaccinated children and their household contacts than the other studies. This may reflect the fact that the study was conducted in younger children with recurrent respiratory tract infections (who comprise a high-risk group for antimicrobial prescribing), the fact that the study was also able to assess the indirect effect of vaccination on other family members by preventing transmission, or both. Of the remaining three studies in children, reductions of 12.6% to 18.6% was reported, though in one of these studies (Salleras *et al.*) the reduction was not significant after adjustment by logistic regression analysis (Table 2). In the two RCTs, the children received two doses of vaccine, and in the other two studies, children received a single vaccination, with only a few exceptions.

**Table 2.** Influenza vaccination studies examining antimicrobial use in children.

Reference (SIGN)	Design	Population	N*	Mean age (range)*	Influenza Season	Outcome description	Outcome			Direction effect (−, −/+, +)
							Measure	Outcome	95% CI/*p*-value	
Esposito (++) [17]	RCT	Children	64/63	3.8 yrs (6 mo–14 yrs)	2000–2001	Antimicrobial prescriptions for URI	VE	44%	<.0001	+
Marchisio (+) [23]	RCT	Children	90/90	2.1/2.2 yrs (1–5 yrs)	2006–2007	Antimicrobial courses	VE	13.2%	<.001	+
Salleras (+) [27]	Non-randomized	Children	1951	NR (3–14 yrs)	2004–2005	Antimicrobial consumption	VE	18.6%	−4.2%−36.4%	−/+
Hardelid (o) [34]	Self-controlled case series	Children	15,543	NR (2–3 yrs)	2013–2014	Amoxicillin prescriptions	VE	12.6%	6.7%−18.2%	+
			22,665	NR (2–4 yrs)	2014–2015	Amoxicillin prescriptions	VE	14.5%	9.6%−19.2%	+

*Vaccinated/unvaccinated; CI: confidence interval; mo: months; NR: not reported; RCT: randomized controlled trial; SIGN: Scottish Intercollegiate Guidelines Network; URI: upper respiratory infection; URI: upper respiratory infection; VE: vaccine effectiveness; yrs: years.

Of the studies that reported on influenza vaccination and antimicrobial use in adults, one study from Japan described the number of antibiotic days in older individuals [28], two studies described antimicrobial use in adult pilgrims to the Hajj [29,32] and one study described antimicrobial use in U.S. students [30]. These studies are summarized in Table 3. In the study conducted by Hara *et al.* in elderly patients hospitalized in the long-term care unit of a Japanese hospital, influenza vaccination reduced the number of days of parenteral antimicrobial use (66% reduction), but for oral antimicrobials the 44% reduction observed was non-significant when looking at the study population as a whole, with a more marked reduction in bedbound patients who can reasonably be assumed to be more frail [28]. Both studies of effectiveness of influenza vaccination in Hajj pilgrims – originating from Malaysia or Pakistan found significant reductions in antimicrobial use of 41% and 66% respectively [29,32]. A similar reduction of 46% (after adjustment) in antimicrobial use associated with influenza-like illness was reported among full-time students attending a U.S.-based college and university [30].

**Table 3.** Influenza vaccination studies examining antimicrobial use in adults.

Reference (SIGN)	Design	Population	N	Mean age (range) in years	Season	Outcome description	Outcome			Direction effect (−, −/+, +)
							Measure	Outcome	95% CI/*p*-value	
Hara (+) [28]	Non-randomized	Elderly	237	80.4 (51–101)	Jan–Mar 1999	Number of days of antimicrobials – oral	Decrease*	42%	.10	−/+
						Number of days of antimicrobials – injected	Decrease*	66%	<.001	+
Mustafa (+) [29]	Non-randomized	Adults	1616	NR	Feb–Mar 2000	Received antimicrobials	VE	66%	54%–75%	+
Nichol (+) [30]	Non-randomized	Students	12,796	25.2/23.3	2002–2006	Antimicrobial use	OR	0.54	0.32–0.90	+
Qureshi (−) [32]	Non-randomized	Adults	2070	45	Feb–Apr 1999	Antimicrobial use	VE	41%	28%–52%	+

*Number of days (standard deviation) of antimicrobials – oral: vaccinated: 2.32 (4.60) *versus* unvaccinated 3.98 (7.35) and number of days of antimicrobials – injected: vaccinated: 2.55 (5.55) *versus* unvaccinated 7.52 (11.2); CI: confidence interval; NR: not reported; OR: odds ratio; SIGN: Scottish Intercollegiate Guidelines Network; VE: vaccine effectiveness.

Three studies, summarized in Table 4, looked at the effect of influenza vaccination of either children or adults on antimicrobial use among household members. In an Italian study, influenza vaccination of infants with a history of recurrent respiratory tract infections was associated with a reduction of 27% and 33% in antimicrobial prescriptions for their parents and siblings, respectively [17]. In a placebo-controlled study of vaccination among children attending U.S. Navy-affiliated day care centres, a dramatic reduction (88%) in the number of antimicrobial prescriptions for their household contacts 5–17 years old was observed [24]. However, there was no significant effect on prescribing for adult household members or siblings younger than 5 years [24]. An evaluation of the effectiveness of vaccination of other family members to prevent influenza in infants (the so-called “cocooning” strategy) found that postpartum influenza vaccination of mothers – but not other household members – was associated with significantly less (45.4% reduction) antimicrobial administration in infants [21].

**Table 4.** Influenza vaccination studies examining transmission within households.

Reference (SIGN)	Design	Population	*N*	Mean age (range)* in years	Season	Outcome description	Outcome			Direction effect (−, −/+, +)
							Measure	Outcome	95% CI/ *p*-value	
Antimicrobial use of household contacts of vaccinated children										
Esposito (++) [17]	RCT	Parents	254	36.8/38.1	2000–2001	Antimicrobial prescriptions	VE	27%	.01	+
		Siblings	95	5.3/5.0	2000–2001	Antimicrobial prescriptions	VE	33%	.01	+
Hurwitz (+) [24]	RCT	Household contacts	29	0–4	1996–1997	Antimicrobial prescriptions	VE	NR		−/+
			59	5–17	1996–1997	Antimicrobial prescriptions	VE	88%	.02	+
			140	≥18	1996–1997	Antimicrobial prescriptions	VE	NR		−/+
Vaccination of household contacts and antimicrobial use in infants										
Maltezou (++) [21]	Non-randomized	Mother–infant	530	30.5 (15–46)/ 30.9 (15–45)	2012–2013	Antimicrobial administration	Difference**	45.4%	.014	+
					2012–2013	Antimicrobial administration	OR***	0.472	0.911–0.245/.025	+
		Other household contacts	1291*	NR	2012–2013	Antimicrobial administration	NR		NS	−/+

* Overall, there were 1844 members in the 553 studied households, including 553 mothers, 525 fathers, 358 siblings, 323 grandparents, 73 other relatives, and 12 caregivers; ** Number of antimicrobial administrations (95% CI) in infants of vaccinated mothers: 22 (4.9–11.6) *versus* infants of unvaccinated mothers: 40 (10.8–19.5);*** The odds ratio is presented here has been calculated in conventional format as the risk in post-partum vaccinated mothers, rather than the original format which presented the risk in those unvaccinated; CI confidence interval; NR: not reported; NS: not significant; OR: odds ratio; RCT: randomized controlled trial; SIGN: Scottish Intercollegiate Guidelines Network; VE: vaccine effectiveness.

Finally, a slightly different approach was described in an ecological study by Kwong and colleagues of influenza-associated respiratory antimicrobial prescriptions before and after universal immunization against influenza was introduced in Ontario. The study found a reduction (after adjustment) of 64% (95% CI 51%–74%), when comparing rates in Ontario to other provinces that targeted vaccination only to at-risk groups [31]. While the magnitude of the observed reduction is comparable to that reported in the adult vaccination studies analysed here, the different study design makes direct comparison problematic.

### 3.2. Pneumococcal vaccination

Of the studies included in this analysis, the vast majority (12/13) analysed vaccination and antimicrobial use in children [18,19,22,25,26,35–41]; the remaining article reported on antimicrobial use in all age groups, though the greatest effects were seen in children [42]. This reflects the fact that unlike influenza, pneumococcal conjugated vaccines (PCVs) are primarily recommended for use in children. As was the case for the influenza-related studies discussed above, study endpoints differed, with antimicrobial use variously presented as whether or not antimicrobial treatment was used, number of antimicrobial courses, prescriptions, courses, or purchases per patient, or as antimicrobial-days for upper respiratory infections (URIs), lower respiratory infections (LRIs) and/or otitis media (OM). Studies in children which could be assigned a SIGN score (RCTs and a prospective cohort study) are summarized in Table 5, while the results from observational studies are summarized in Table 6. An additional complicating factor is the fact that while seasonal influenza vaccines are all based on the same antigens, selected in advance for that season’s predicted dominant strains, there are several PCVs on the market [43]. Although clinical data suggest the most commonly used vaccines have similar effectiveness with regard to overall prevention of pneumococcal disease, this remains a potential source of difference between studies [43]. For the studies reported here, nine reported results that included the 7-valent PCV7 vaccine, five reported results that included the 10-valent pneumococcal *Haemophilus influenzae* protein D conjugate vaccine (PHiD-CV10), five reported results that included the 13-valent PCV13 vaccine, one from the experimental 9-valent PCV9 vaccine and one from the 23-valent non-conjugated vaccine. The sum of these numbers are greater than the total number of studies included in this analysis, because several studies reported results spanning periods where different pneumococcal vaccines were in use. Finally, the duration of follow-up varied widely in studies of the impact of pneumococcal vaccination. In studies where both influenza and pneumococcal vaccines were assessed simultaneously, or in clinical trials of pneumococcal vaccination, the follow-up period at 1–3 years was comparable to that of the influenza trials. However, the population cohort studies for pneumococcal vaccination typically spanned the period before and after vaccination introduction, leading to data accumulation over 5–14 years, which may hamper the assessment of the impact of pneumococcal vaccination on antibiotic use, given the downward trend driven by guideline changes [35,42].

**Table 5.** Pneumococcal vaccination studies examining antimicrobial use in children: RCTs and non-randomized cohort studies.

Reference (SIGN)	Design	Population	*N*	Mean age (range)*	Vaccine	Outcome description	Outcome			Direction effect (−, −/+, +)
							Measure	Outcome	95% CI/p-value	
Palmu (++) [19]	RCT	Infants/children	30,527	NR	PHiD-CV10	Antimicrobial treatment	VE	7%	0%−14%	+
Dagan (++) [18]	RCT	Children	131/130	28 mo	PCV9	Antimicrobial days for URI	RR	0.90	0.84–0.97/.005	+
						Antimicrobial days for LRI problems	RR	0.53	0.45–0.62/<.001	+
						Antimicrobial days for OM	RR	0.80	0.74–0.86/<.001	+
						Antimicrobial days for Others	RR	1.01	0.87–1.16/0.918	−/+
Esposito (++) [22]	Non-randomized	Infants	811/744	82 days** (75–105)	PCV7	Antimicrobial courses	RR	0.89	0.83–0.94/.0001	+
				6–12 mo		Antimicrobial courses	RR	1.01	0.89–1.14/.87	−/+
				13–18 mo		Antimicrobial courses	RR	0.86	0.76–0.95/.004	+
				19–24 mo		Antimicrobial courses	RR	0.89	0.79–1.01/.06	−/+
				25–30 mo		Antimicrobial courses	RR	0.78	0.67–0.90/.0008	+
O’Grady (+) [26]	RCT	Children	31/30	6.8 yrs (NR)	PHiD-CV10	Antimicrobial courses: <14 days post-dose 2	Risk difference	−1.1/100 weeks	−2.6–0.1	−/+
						<28 days post-dose 2	Risk difference	−0.7/100 weeks	−2.4–1.1	−/+
						<14 days post-dose 2	IDR	0.81	0.61–1.09	−/+
						<28 days post-dose 2	IDR	0.87	0.66–1.15	−/+
Fireman (+) [25]	RCT	Infants/children	18,926/18,942	NA	PCV7	Antimicrobial prescriptions	Difference***	5.4%	4.0%–6.7%	+

*Vaccinated/unvaccinated; **Median; ***Reduced antimicrobial prescriptions in all follow-up starting at Dose 1; CI: confidence interval; IDR: incidence density ratio; LRI: lower respiratory infection; mo: months; NR: not reported; OM: otitis media; OR: odds ratio; PHiD-CV10: pneumococcal *Haemophilus influenzae* protein D conjugate vaccine; PCV7: heptavalent pneumococcal conjugate vaccine; PCV9: 9-valant pneumococcal conjugate vaccine; RCT: randomized controlled trial; RR: relative risk; SIGN: Scottish Intercollegiate Guidelines Network; URI: upper respiratory illness; VE: vaccine effectiveness.

**Table 6.** Pneumococcal vaccination studies examining antimicrobial use in children: observational studies.

Reference	Design	Population	*N*	Age	Vaccine	Outcome description	Outcome						Direction effect (−, −/+. +)
							Measure		Outcome		95% CI/ *p*-value		
Eythorsson [35]	Population-based	Birth cohort – children	50,570	<3 yrs	PHiD-CV10	Null antimicrobial prescriptions	IRR		1.16		1.10–1.23		+
					PHiD-CV10	1–4 antimicrobial prescriptions	IRR		1.08		1.06–1.11		−
					PHiD-CV10	5–9 antimicrobial prescriptions	IRR		0.92		0.89–0.95		+
					PHiD-CV10	10–14 antimicrobial prescriptions	IRR		0.77		0.72–0.82		+
					PHiD-CV10	≥15 antimicrobial prescriptions	IRR		0.83		0.74–0.93		+
				0–5 mo	PHiD-CV10	Antimicrobial prescriptions	IRR		0.82		0.79–0.85		+
				6–11 mo	PHiD-CV10	Antimicrobial prescriptions	IRR		0.84		0.83–0.86		+
				12–17 mo	PHiD-CV10	Antimicrobial prescriptions	IRR		0.93		0.91–0.94		+
				18–23 mo	PHiD-CV10	Antimicrobial prescriptions	IRR		0.94		0.92–0.96		+
				24–29 mo	PHiD-CV10	Antimicrobial prescriptions	IRR		0.87		0.85–0.89		+
				30–35 mo	PHiD-CV10	Antimicrobial prescriptions	IRR		0.89		0.86–0.91		+
Fortanier [36]	Population-based	Infants	255,154	<2 yrs	PCV7/PCV10**	Antimicrobial purchases	RR		0.984		0.977–0.992		+
Howitz [37]	Population-based	Children	1.04–1.08 million	0–15 yrs	PCV7	Antimicrobial prescriptions	IRR		0.9542		0.9536–0.9548		+
				0–15 yrs	PCV13	Antimicrobial prescriptions	IRR		0.9048		0.9043–0.9052		+
Johansson Kostenniemi [42]	Population-based	Whole population	259,183	All ages	PCV7/PCV13	Antimicrobial prescriptions	Decrease#		NR		–		+
				All ages	PCV7/PCV13	Antimicrobial Prescription for URI	Decrease#		37.1%		NR		+
				0–4 yrs	PCV7/PCV13	Antimicrobial Prescription for pneumonia	Decrease#		28.6%		NR		+
Kinlaw [38]	Population-based	Birth cohort- infants	561,729	≤1 yr	PCV7/PCV13	At least one antimicrobial prescription	1-year risk		4.4%		3.4% to 5.5%		+
Lau [39]	Population-based	Children	567,275	<10 yrs	PCV7/PCV13	Antimicrobial prescriptions for OM	Decrease***		72.9%		–		+
					PCV7	Antimicrobial prescriptions for OM	ITS analysis		18.9%		16.0%−21.7%		+
					PCV13	Antimicrobial prescriptions for OM	ITS analysis		12.2%		8.6%−15.6%		+
Palmu [40]	Population-based	Birth cohort- Children	NR	<4.5 yrs	PCV10	Antimicrobial prescriptions	RR		17.5%		17.0%−18.1%		+
Zhou [41]	Population-based	Infants	20,628/153,812*	<2 yrs	PCV7	Antimicrobial prescriptions for AOM	Decrease		41.9%		–		+
							Absolute difference		522		513–530 <.001		+

*1997: *N* = 20,628; 2004: *N* = 153,812; **PCV7 compared with PCV10; *** 2002 *versus* 2012; # results presented in text; AOM: acute otitis media; CI: confidence interval; IRR: incidence rate ratio; mo:months; NR: not reported; OM: otitis media; PHiD-CV10: pneumococcal Haemophilus influenzae protein D conjugate vaccine; PCV7: heptavalent pneumococcal conjugate vaccine; PCV10: 10-valent pneumococcal conjugate vaccine; PCV13: 13-valent pneumococcal conjugate vaccine; RR: rate ratio; SIGN: Scottish Intercollegiate Guidelines Network; URI: upper respiratory illness; yr(s): year(s); ITS: Interrupted time series.

All of the RCTs and prospective studies included were considered at low or moderate risk of bias and the results for the pneumococcus-vaccinated cohorts as a whole suggest a significant overall reduction in antimicrobial prescribing in the range of 5–17% (Table 5 and [18,19,22,25,26,40]). However, while the sub-analyses showed a clear trend towards significant reduction of antimicrobial use in vaccinated children and infants, the magnitude of the effect was quite different in different sub-groups. The analysis of PCV9 effect by Dagan and colleagues in Israeli children attending day-care showed that vaccination reduced overall antimicrobial days by 17%. This benefit was attributed to reductions in prescribing for URI (reduced 10%), OM (reduced 20%) and LRI (reduced 47%). There was no reduction in antimicrobial days for other illnesses [18]. When stratified for age, children under 36 months of age saw benefits in terms of reduced antimicrobial use for OM and LRI, while children 36 months and older saw benefits in terms of reduced antimicrobial use prescribed for URI and LRI [18]. Three studies in vaccinated infants, drawing on populations from Finland, Italy and the USA, using PCV7 and PHiD-CV10, also found significant reductions in antimicrobial treatment (Table 5 and [19,22,25]). Although the endpoints in the three studies are different enough that a direct comparison is not possible, it is safe to say that reduction in prescribing associated with OM appeared to be one of the major drivers, consistent with the important role pneumococci play in the aetiology of this disease.

The final RCT discussed here is significantly different in terms of target population to those discussed above, since it assessed impact of the PHiD-CV10 vaccine in children and adolescents under 18 years of age with recurrent protracted bacterial bronchitis, chronic suppurative lung disease or bronchiectasis. Although the vaccinated participants were less likely to have respiratory symptoms and required fewer short-course antimicrobial treatments than the control group, this difference was not significant (incidence density ratio 0.81, 95% confidence interval 0.61, 1.09), possibly because in this high-risk group, antimicrobial use (also for unrelated infections) was high throughout the trial [26].

The remaining studies evaluated here (Table 6) are all database or register studies comparing incidence before and after vaccination [35–37,39–42]. Despite these similarities, there remain substantial differences in endpoints, duration of the studies and the vaccines used, so direct comparisons of the results need to be approached with caution. That said, certain trends are immediately discernible. For example, all of the studies found a significant reduction in the percentage of children prescribed antimicrobials after the introduction of universal childhood vaccination with PCVs (Table 6) [19,35–37,39–42]. In addition, where this was analysed, the data suggest that there was a significant reduction in the percentage of children requiring recurrent antimicrobial prescriptions, as well as in the volume of prescribing [35]. As was reported for the trials discussed and summarized in Tables 5 and 6, significant reductions in antimicrobial prescribing for OM, pneumonia and respiratory tract infections appeared to be associated with all of the PCVs tested, suggesting that this is a class effect, consistent with the reduction in antimicrobial use being driven by reduction in the incidence of pneumococcal disease. However, the magnitude of the reported reductions ranged from a few percent to over 73% (Table 6). It is unlikely that differences in the precise endpoint can explain this degree of variation, and instead suggest the influence of major methodological differences or confounders.

### 3.3. Combined influenza and pneumococcal vaccination

Two studies were identified that examined the potential effect of vaccinating against both influenza and *pneumococcus*. One was a double-blind, placebo-controlled RCT in children of 18–72 months of age, with a previous diagnosis of respiratory tract infection, conducted in the Netherlands over two influenza seasons, with a follow-up period of 18 months [20]. The other was a cohort study from France, drawing on regional health records of adults 65 years and older [33]. Study parameters are defined in Table 7. In the RCT in children, trivalent influenza vaccine (TIV) or TIV + PCV7 vaccinations were associated with only non-significant reductions in the percentage of children receiving an antimicrobial prescription. There were also no significant differences between children receiving influenza vaccination plus placebo, *versus* influenza vaccination plus PCV7. The authors note that results may reflect, at least in part, the mild influenza seasons in the years studied, the short (18 month) observation period, and the presence of suboptimally matched H3N2 strain and influenza B viruses in the 2003–2004 and 2004–2005 influenza seasons, respectively [20].

**Table 7.** Influenza plus pneumococcal vaccination studies examining antimicrobial use.

Reference (SIGN)	Design	Population	*N*	Median Age (range)	Season	Vaccine	Outcome description	Outcome			Direction effect (−, −/+, +)
								Measure	Outcome	95% CI/ *p*-value	
Jansen (++) [20]	RCT	Children	579*	3.0 yrs (18–72 mo)	2003–2005	TIV + PCV7	Antimicrobial prescriptions during influenza season	IRR	0.73	0.40 to 1.32	−/+
			148	3.1 yrs (18–72 mo)	2003–2005	TIV + placebo	Antimicrobial prescriptions during influenza season	IRR	0.89	0.50 to 1.61	−/+
Mahamat (−) [33]	Non-randomized	Elderly	68,897	75.2 yrs (65–102 mo)	Oct–Dec 2004	TIV + PPV23, TIV, PPV23 or none	Antimicrobial consumption	Difference**	–	NS	−/+

*N = 163,148 and 160 in TIV/PCV7, TIV + Placebo and Placebo-only groups respectively; **Not quantifiable, data presented in figures; CI: confidence interval; IRR: incidence rate ratio; mo: months; NS: not significant; PCV7: heptavalent pneumococcal conjugate vaccine; PPV23: 23-valent pneumococcal capsular polysaccharide vaccine; RCT: randomized controlled trial; SIGN: Scottish Intercollegiate Guidelines Network; TIV: trivalent influenza vaccine; yrs: years.

The observational study in older adults, conducted over two influenza seasons in France, compared results in older adults who had been offered influenza vaccination and pneumococcal vaccination (the 23-valent pneumococcal capsular polysaccharide vaccine: PPV23) as part of a vaccination campaign [33]. Only 62.5% adults received vaccination, and not all those vaccinated received both vaccines, allowing a comparison to be made between cohorts who had received both, either, or neither vaccine. It should be noted, however, that a relatively small percentage (5.5%) received the pneumococcal vaccination alone, making this arm of the study potentially less robust. Antibiotic use peaked in all groups immediately after the influenza season in both years studied, suggesting at least a temporal association, but interestingly, those who had received both vaccines also had the highest rate of antimicrobial prescribing, while those who received neither vaccine also had the lowest rate of antimicrobial prescribing. Influenza vaccination (with or without PPV23) significantly reduced all-cause mortality in this study, indicating that the vaccine appeared to be effective, but no reduction in antimicrobial prescribing was reported. This raises the concern (also noted by the authors) that there may be a selection bias, possibly reflecting access to healthcare services or prioritization of different groups for vaccination.

## 4. Discussion

The starting point for this review was the growing concern over increasing levels of AMR [4–6], and an interest in what concrete actions might be taken to improve antimicrobial stewardship at a global level. One of the starting assumptions was that for any intervention to be broadly taken up, it needed to be practical and economically sustainable, and it needed to be supported by sufficient data to enable its utility to be assessed. We hypothesized that the use of existing vaccinations could reduce disease and therefore, indirectly to reduce antimicrobial prescribing. If this was correct, vaccination could potentially meet the first two criteria, but that data describing the potential magnitude of the effect was not readily accessible, being scattered across multiple publications, each – on its own – with limited generalizability. An example of this is the publication of an analysis of the paediatric pneumococcal vaccination programme in Finland, which attempted to outline the downstream benefits of vaccination, which include reduced healthcare utilization and reduced antimicrobial prescribing [19]. While the effect is measurable in that case, it is not clear if it would apply to other populations or other settings, a point the authors themselves make [19]. We therefore conducted a systematic review to assess the potential impact of vaccination on antimicrobial prescribing.

The studies summarized in this review appear to confirm the starting hypothesis that vaccination with trivalent influenza vaccines and conjugated pneumococcal vaccines significantly reduces antimicrobial consumption in the period immediately or shortly after vaccination. Of the 26 studies retrieved for this analysis, 23 found significant reductions in antimicrobial use in vaccinated individuals or groups, and in the remaining 3, all but one found non-significant reductions. Further, all of the studies but one rated as being at low risk of bias using the SIGN scoring method found significant reductions in antimicrobial use. The one study considered at low risk of bias, that did not find a significant difference noted potential epidemiological considerations that may have blunted the effect of vaccination – specifically a poor match between the influenza vaccine used and common circulating strains in the influenza seasons during the study period [20]. Nonetheless, the data strongly suggests that vaccination against either influenza or pneumococci can reduce overall healthcare visits and antimicrobial consumption and sharply reduce antimicrobial prescribing in patients with diagnoses associated with otitis media and upper respiratory tract infections [13–17,34], although the variations in study design, vaccines used and study populations make it impossible to provide a single precise estimate of the impact of vaccinating against influenza or pneumococci.

However, despite the impossibility of synthesizing a single figure, some useful observations may be made. In children, influenza vaccination appeared to reduce overall antimicrobial consumption by approximately 14.5% (median value, unadjusted), albeit with significant variation [23,27,34]. This is a conservative estimate, as it looks only at vaccine effectiveness in the vaccinated children: household transmission studies suggest an additional significant benefit from reduced transmission [17,24]. In adults, a substantially larger proportional reduction in antimicrobial consumption after vaccination was seen, with an unadjusted median of 52% across the studies included in this review. This may reflect the relatively higher likelihood in adults (compared to young children) that influenza will provoke symptoms severe enough to cause the infected individual to seek medical advice (and therefore potentially be prescribed antimicrobials) [44–46].

For pneumococcal vaccination, the situation is similar – there are sharp declines in antimicrobial use associated with specific diagnoses associated with pneumococcal infection such as OM, ranging from 21% to 73% [18,19,22,25,26,35–41]. Smaller, but still significant declines in total antimicrobial prescribing in the range 2–19% were reported [18,19,22,25,26,35–41]. While this reduction may not seem dramatic, the total number of prescriptions associated with diagnoses of pneumococcal infection is such that this could result in significant savings in antimicrobial utilization – it has been estimated in one study that one antimicrobial prescription is avoided for every 5 children vaccinated against pneumococci [19]. That said, however, it must be acknowledged that there are significant gaps in the literature, and that any conclusions on the magnitude of the reduction and the general applicability of these findings must be approached with caution.

This review is subject to several important limitations: the first is that very little published data is available on vaccines for pathogens other than influenza or pneumococci, as noted in the methodology section. Any current analysis therefore, can only hope to give a partial picture of the potential overall impact of vaccination for reducing antimicrobial use.

A second major limitation is that there was no consensus between the reported studies on how antimicrobial use was measured and reported. The studies included in this review use different measures, such as antimicrobial-days, number of prescriptions, or binary measures such as whether antimicrobials were dispensed or not. Some studies have measured any antimicrobial, others have analysed by type. The disparity of measurements used could potentially lead to different outcomes and may explain the high degree of variation reported: for example, a reduction in antimicrobial-days required may detect a reduction where a binary analysis of whether any antimicrobial was used or not, may fail to do so. These differences in reported outcome prevent the synthesis of the collected data into a single value, and the analysis here is therefore restricted to indicating the general magnitude and range of the impact observed.

The third limitation is that the majority of the published studies assessed in the review focus on specific risk groups, such as infants or older adults with comorbidities. While the rationale for this is obvious – these are priority groups for vaccination – it does mean that our ability to generalize from these results is limited. Against this limitation however, we can balance the observation that reductions in antimicrobial use among at least some of the vaccinated study populations are reported by the majority (23 out of 26) of the studies included in this review.

One final limitation is that most of the studies reported here are from high-income countries where antimicrobial prescribing is relatively strictly controlled. Access to antimicrobials varies significantly between countries [2] and it is not clear if quantitatively similar reductions could be obtained in low- and middle-income settings, especially those where antimicrobials are available without a prescription. There is however, reason to think that this may be the case. Two studies in Hajj pilgrims, drawing on subjects from Pakistan and Malaysia, found significant reductions in the use of antimicrobials, on a scale (41% and 61%, respectively) comparable to that reported in studies from high income countries [29,32]. Both studies also reported a reduction of similar magnitude in the dispensing or use of non-prescription remedies [29,32]. These data are therefore consistent with the underlying assumption presented in the introduction, that reductions in disease (in this case, influenza-like illness) also result in a reduction in treatment-seeking generally, and thus, indirectly, in consumption of medicines, including antimicrobials. Hypothetically, this pattern of behaviour should, be generally applicable. It should be noted, however that these studies were non-blinded with regard to the participants’ vaccine status and thus possibly subject to bias with regard to treatment-seeking or treatment-dispensing behaviour.

While the studies under review that reported disease incidence all found significant decreases in the outcome measured, the association of decreased mortality with declines in antimicrobial use did not hold in every case. In one large observational study in adults aged 65 and older from France, it was shown that influenza vaccination was associated with significantly reduced mortality in vaccinees, particularly in those who also received concurrent PPV23 vaccination [33]. However, no associated reduction in antimicrobial prescribing was observed. This is particularly puzzling, since in all groups antimicrobial prescribing rose through the influenza season, peaking shortly after the peak of the influenza season and then falling. This is consistent with the concept that antimicrobials were being prescribed for patients based on respiratory infections, but the reduction in mortality is not reflected in prescribing numbers. The authors of that study suggest that this may be due to the fact that in older adults, the effectiveness of the influenza vaccine against hospitalization and death is generally higher than its effectiveness at preventing influenza-like illness, so that vaccinated individuals may still seek healthcare, and be prescribed antimicrobials even if the severity of their illness – and consequently, mortality – is reduced [33]. It is, however, not possible to confirm that hypothesis from the data available.

The health status of vaccinees or contacts may have also confounded results in some studies. In one U.S. study looking at antimicrobial use within families with children attending day-care, vaccination against influenza of the children was associated with reduced morbidity in unvaccinated siblings, and reduced use of both antimicrobials and non-prescription medicines [24]. However, no effect on either aspect was seen on adult contacts. In contrast, a similar study in Italy found reduced morbidity and antimicrobial use in both parents and siblings of vaccinated children [17]. It is possible that this may reflect differences in the tendency to seek healthcare for child and adult patients with influenza-like illness, but the authors of the U.S. study suggested that the lack of effect they observed in adults may reflect the relatively high proportion of adults in their study population who were vaccinated against influenza [24]. It may also reflect the small numbers vaccinated in some age cohorts [24].

The last limitation that needs to be addressed is the challenge of attributing changes in antimicrobial prescribing over time to vaccination. While this is a less significant issue in RCTs, many of the studies discussed here are observational studies, some of which have collated data over extended periods. Changes in diagnostic criteria or prescribing guidelines following heightened awareness about the threat of antimicrobial resistance [35,42], healthcare seeking patterns, other diseases, such as the pandemic influenza outbreak [37], availability of specific antimicrobial formulations [35] or even factors that affect disease incidence, but which are unrelated to vaccination (for example, a decrease in OM associated with a decrease in second-hand smoke exposure [36,42,47]) may all impact antimicrobial use. While this can be controlled for to some extent, for example by comparing antimicrobial prescribing rates to specific diagnoses, or to antimicrobial use for conditions unrelated to the vaccine-preventable disease, the risk of unidentified confounders cannot be excluded.

Bearing these caveats in mind, data from 9 out of 10 of the influenza vaccination studies reported significant reductions in at least one of the measures of subsequent antimicrobial prescribing used (Tables 2–4). Although there were some outliers, reported vaccine effectiveness at reducing antimicrobial prescribing in different groups tended to be relatively consistent within age cohorts. The different outcome measures reported preclude the synthesis of this data into a formal point estimate, but in children, effectiveness of influenza vaccination (Table 2) showed a median reduction of 14.5%, range 12.6–44.0%) while in adults (Table 3) the median reduction was 64%, with a range of 41–66%. Studies involving both adults and children reported more variable outcomes (Table 4: median reduction 33%, range 0–88%, depending on how age cohorts were stratified). Encouragingly, these conclusions are similar to a very recent systematic review of vaccination impact on antibiotic use despite the fact that the two reviews used different inclusion criteria and reviewed an overlapping, but not identical list of publications [48].

With regard to pneumococcal vaccination, all but one of the 13 studies reviewed were conducted in infants or children and among this group. These studies are subject to the same caveats regarding diversity of outcome measures, study parameters, plus an additional caveat regarding the valency of the different vaccines used, which again precluded the synthesis of results. Nonetheless, 12 out of 13 studies found a significant reduction in antimicrobial use in at least one of the reporting measures used (Tables 5 and 6). The median reduction across these different measures was 13.5%, with a range of 1.6–37.1%. The majority of studies reported reductions in overall prescribing in the range of 5–20% (Tables 5 and 6) with much higher percentage reductions in prescribing associated with acute otitis media. Interestingly, the two studies which looked at a combination of influenza and pneumococcal vaccination did not show an additional reduction in antimicrobial use, even though the vaccinations were clearly effective against both influenza and pneumococcal-related disease and showed an additive effect on mortality in older adults (Table 7). This could reflect the fact that outcomes in both studies were measured across influenza seasons, which may have biased the results towards that attributable to influenza-like illness, or it may reflect undetected biases in healthcare access or patient population as discussed in the Results section [20].

To conclude, the published data appear to be conclusive that vaccination against influenza and pneumococcal disease has the potential to significantly reduce antimicrobial consumption. While this supports the initial hypothesis we set out to test, it is still only a partial answer. The lack of studies on the impact of other vaccines, such as those for rotavirus or *Neisseria meningitidis* infections suggests that the total impact of vaccination may be significantly greater than that outlined here, and more research to clarify this aspect is urgently needed. In addition, while it is intuitive that reduced antimicrobial use will translate into reduced antimicrobial resistance, it is not formally proven that this is the case, although recent reviews suggest that use of conjugate pneumococcal vaccines reduces carriage and transmission of resistant pneumococcal strains [49,50] as well as the effect on total prescribing. If the proportion of second line antimicrobials used to treat patients is used as proxy for treatment failure and AMR, then there is already evidence suggestive of a benefit [35–37,39–42,51] but in this area too, more research will be required.

Finally, it must be noted that although the data appear to strongly support the ability of influenza and pneumococcal vaccination to reduce overall antimicrobial prescribing, there remain significant gaps. In particular, there is very little data on effectiveness in older adults, compared to that in infants and children. Given the high attack rates for both influenza and pneumococcal disease in this age group, this is a serious omission. The published literature as reviewed here is also very heavily biased in favour of studies from North America and Northern Europe. Whether the observed reductions can be duplicated in other settings remain unknown, though the fact that two studies on influenza from outside this region showed similar reductions in antimicrobial use is encouraging [29,32]. Studies to address these questions, as well as improved health outcome models that include potential benefits from reduced antimicrobial prescribing are urgently needed. Nonetheless, these gaps should not be taken as an excuse for inaction. The existing data clearly support the initial hypothesis that reducing symptomatic disease by vaccination against influenza and *pneumococcus* can also reduce antimicrobial prescribing. This makes it one of the few tools we have in hand that has proven effectiveness for antimicrobial stewardship and one that can be put into practice today – both factors that should drive healthcare systems to prioritize increasing uptake of these vaccines.

## Supplementary Material

Supplemental Material
