# Supplementary material for: Effect of vaccination on the use of antimicrobial agents: a systematic literature review
Source: Ann Med. 2020 Jun 29;52(6):283–99. doi: 10.1080/07853890.2020.1782460 (PMC7880080; doi:10.1080/07853890.2020.1782460)
Supplement: Supplemental Material [file IANN_A_1782460_SM9656.zip › Supplemental files/Doherty_AMR vaccination_SupMat1.docx]

# Supplementary Material 1. SIGN Checklists

| **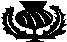 S I G N** | | **Methodology Checklist 2: Controlled Trials** | | | | |
| --- | --- | --- | --- | --- | --- | --- |
| Study identification (*Include author, title, year of publication, journal title, pages*) | | | | | | |
| Guideline topic: | | | Key Question No: | | Reviewer: | |
| **Before** completing this checklist, consider:   1. Is the paper a **randomised controlled trial** or a **controlled clinical trial**? If in doubt, check the study design algorithm available from SIGN and make sure you have the correct checklist. If it is a **controlled clinical trial** questions 1.2, 1.3, and 1.4 are not relevant, and the study cannot be rated higher than 1+ 2. Is the paper relevant to key question? Analyse using PICO (Patient or Population Intervention Comparison Outcome). IF NO REJECT (give reason below). IF YES complete the checklist. | | | | | | |
| Reason for rejection: 1. Paper not relevant to key question □ 2. Other reason □ (please specify): | | | | | | |
| **Section 1: Internal validity** | | | | | | |
| ***In a well conducted RCT study…*** | | | | 1. ***Does this study do it?*** | | |
| 1.1 | The study addresses an appropriate and clearly focused question. | | | Yes □  Can’t say □ | | No □ |
| 1.2 | The assignment of subjects to treatment groups is randomised. | | | Yes □  Can’t say □ | | No □ |
| 1.3 | An adequate concealment method is used. | | | Yes □  Can’t say □ | | No □ |
| 1.4 | The design keeps subjects and investigators ‘blind’ about treatment allocation. | | | Yes □  Can’t say □ | | No □ |
| 1.5 | The treatment and control groups are similar at the start of the trial. | | | Yes □  Can’t say □ | | No □ |
| 1.6 | The only difference between groups is the treatment under investigation. | | | Yes □  Can’t say □ | | No □ |
| 1.7 | All relevant outcomes are measured in a standard, valid and reliable way. | | | Yes □  Can’t say □ | | No □ |
| 1.8 | What percentage of the individuals or clusters recruited into each treatment arm of the study dropped out before the study was completed? | | |  | | |
| 1.9 | All the subjects are analysed in the groups to which they were randomly allocated (often referred to as intention to treat analysis). | | | Yes □  Can’t say □ | | No □  Does not apply □ |
| 1.10 | Where the study is carried out at more than one site, results are comparable for all sites. | | | Yes □  Can’t say □ | | No □  Does not apply □ |
| **Section 2: OVERALL ASSESSMENT OF THE STUDY** | | | | | | |
| 2.1 | How well was the study done to minimise bias?  Code as follows: | | | High quality (++) □  Acceptable (+) □  Low quality (-) □  Unacceptable – reject (0) □ | | |
| 2.2 | Taking into account clinical considerations, your evaluation of the methodology used, and the statistical power of the study, are you certain that the overall effect is due to the study intervention? | | |  | | |
| 2.3 | Are the results of this study directly applicable to the patient group targeted by this guideline? | | |  | | |
| 2.4 | **Notes.** Summarise the authors’ conclusions. Add any comments on your own assessment of the study, and the extent to which it answers your question and mention any areas of uncertainty raised above. | | | | | |

| **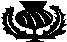 S I G N** | | **Methodology Checklist 4: Case-control studies** | | |
| --- | --- | --- | --- | --- |
| Study identification (*Include author, title, year of publication, journal title, pages*) | | | | |
| Guideline topic: | | | Key Question No: | Reviewer: |
| **Before** completing this checklist, consider:   1. Is the paper really a case-control study? If in doubt, check the study design algorithm available from SIGN and make sure you have the correct checklist. 2. Is the paper relevant to key question? Analyse using PICO (Patient or Population Intervention Comparison Outcome). IF NO REJECT (give reason below). IF YES complete the checklist. | | | | |
| Reason for rejection: Reason for rejection: 1. Paper not relevant to key question □ 2. Other reason □ (please specify): | | | | |
| **Section 1: Internal validity** | | | | |
| ***In an well conducted case control study:*** | | | 1. ***Does this study do it?*** | |
| 1.1 | The study addresses an appropriate and clearly focused question. | | Yes □  Can’t say □ | No □ |
| Selection of subjects | | | | |
| 1.2 | The cases and controls are taken from comparable populations. | | Yes □  Can’t say □ | No □ |
| 1.3 | The same exclusion criteria are used for both cases and controls. | | Yes □  Can’t say □ | No □ |
| 1.4 | What percentage of each group (cases and controls) participated in the study? | | Cases:  Controls: | |
| 1.5 | Comparison is made between participants and non-participants to establish their similarities or differences. | | Yes □  Can’t say □ | No □ |
| 1.6 | Cases are clearly defined and differentiated from controls. | | Yes □  Can’t say □ | No □ |
| 1.7 | It is clearly established that controls are non-cases. | | Yes □  Can’t say □ | No □ |
| ASSESSMENT | | | | |
| 1.8 | Measures will have been taken to prevent knowledge of primary exposure influencing case ascertainment. | | Yes □  Can’t say □ | No □  Does not apply □ |
| 1.9 | Exposure status is measured in a standard, valid and reliable way. | | Yes □  Can’t say □ | No □ |
| CONFOUNDING | | | | |
| 1.10 | The main potential confounders are identified and taken into account in the design and analysis. | | Yes □  Can’t say □ | No □ |
| STATISTICAL ANALYSIS | | | | |
| 1.11 | Confidence intervals are provided. | | Yes □ | No □ |
| **Section 2: OVERALL ASSESSMENT OF THE STUDY** | | | | |
| 2.1 | How well was the study done to minimise the risk of bias or confounding? | | High quality (++) □  Acceptable (+) □  Low quality (-) □  Unacceptable – reject (0) □ | |
| 2.2 | Taking into account clinical considerations, your evaluation of the methodology used, and the statistical power of the study, do you think there is clear evidence of an association between exposure and outcome? | | Yes □  Can’t say □ | No □ |
| 2.3 | Are the results of this study directly applicable to the patient group targeted by this guideline? | | Yes □ | No □ |
| 2.4 | **Notes.** Summarise the author’s conclusions. Add any comments on your own assessment of the study, and the extent to which it answers your question and mention any areas of uncertainty raised above. | | | |
